# Nanochitin as Binder in Li-Ion Battery Anodes Enabling Aqueous Processing and Superior Solid Electrolyte Interphase

**DOI:** 10.1021/acsami.6c01089

**Published:** 2026-04-18

**Authors:** Amritha P. Sandra, Vishnu Arumughan, Roberta Teixeira Polez, Fredrik Lindgren, Maria Hahlin, Han Tao, Monika Österberg, Eero Kontturi, Rakel Wreland Lindström

**Affiliations:** † Department of Chemical Engineering, KTH Royal Institute of Technology, SE-100 44 Stockholm, Sweden; ‡ Department of Bioproducts and Biosystems, School of Chemical Engineering, 174277 Aalto University, Espoo, FI-0076 Aalto, Finland; § Department of Chemistry − Ångström Laboratory, Uppsala University, Box 538, SE-751 21 Uppsala, Sweden; ∥ Department of Physics and Astronomy, Uppsala University, Uppsala 751 20, Sweden

**Keywords:** binders, aqueous processing, chitin nanofibers, lithium-ion battery, graphite, gas evolution

## Abstract

Binders are essential components in battery systems that maintain the electrode structure and integrity throughout cycling. The choice of binder affects processing, electrochemical behavior, and end-of-life recovery. The widely used poly­(vinylidene difluoride) (PVDF) binder is increasingly scrutinized since it is a polyfluoroalkyl compound and requires processing in organic solvents like *N*-methyl-2-pyrrolidone. Consequently, efforts are underway to identify more sustainable options. In this work, we present chitin nanofibers (ChNFs), derived from fisheries waste, as a biobased, fluorine-free alternative that enables fully aqueous electrode fabrication. ChNFs disperse more than 90% of graphite in water without the need for auxiliary agents such as surfactants. Colloidal probe microscopy shows that adhesion between protonated ChNFs and graphite depends on pH, likely being governed by cation−π interactions. This strong affinity facilitates the remarkable dispersing capability of ChNFs through a multifaceted mechanism. Adsorbed nanofibers confer electro-steric stabilization by extension into the aqueous phase. The strong ChNF network can physically entrap larger particles, greatly enhancing the long-term stability of ChNF–graphite suspensions. The ChNF–graphite dispersion exhibits rheological behavior suited for forming uniform electrode coatings. Electrodes prepared with 4% ChNFs deliver specific capacities of 370 mA h g^–1^ and enhanced capacity retention over 100 cycles compared to PVDF-based counterparts. Electron microscopy, X-ray photoelectron spectroscopy, and online electrochemical mass spectrometry analyses reveal that the nature of the binder dictates the solid electrolyte interphase (SEI) characteristics. The ChNF produces a more robust and stable SEI that suppresses electrolyte reduction, directly contributing to the enhanced electrochemical performance.

## Graphical abstract

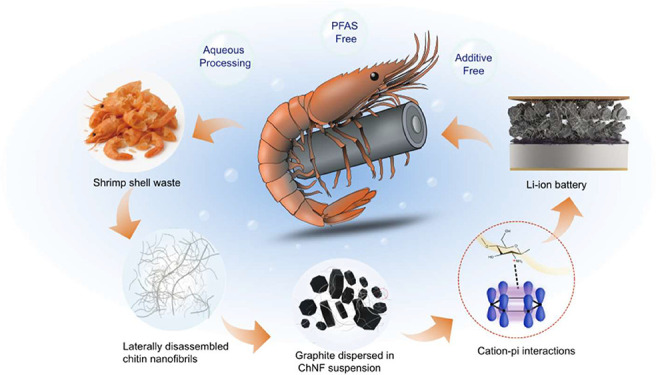

## 1 Introduction

The demand for lithium-ion batteries (LIBs) is consistently growing due to expanding markets for electric vehicles and portable electronic devices.^1,2^ Consequently, developing cost-effective, sustainable, and safe battery technologies is of paramount importance. Recently, significant efforts have been directed toward replacing fossil-based battery components with biobased alternatives, particularly high-volume components such as active carbon materials and separators.^3–6^ Among the battery components, binders are low-weight and electrochemically inactive components, which play a crucial role in forming a stable active material network by facilitating adhesion between the active material, conductive carbon, and the current collector, ensuring smooth electron flow through the electrodes.^7^ Additionally, the binder material is essential for the mechanical stability of electrodes, helping them endure volume changes during cycling, improving the cycling stability, and reducing irreversible capacity loss. Despite binders constituting only 2–5% of the battery electrode by weight, they primarily dictate the electrode processing methodology, electrochemical performance, and recyclability.^8,9^

Currently, poly­(vinylidene difluoride) (PVDF) is the industry standard due to its excellent binding and electrochemical stability. However, PVDF, a fluorinated polymer, presents significant environmental concerns.^10^ The European Union is on the cusp of enforcing stringent regulations on polyfluoroalkyl substances this year, a move expected to trigger the ‘Brussels effect’ and influence material choices globally. PVDF is one of the candidates likely to be affected.^11,12^ Beyond environmental and safety concerns, PVDF processing relies on hazardous and costly organic solvents, such as *N*-methyl-2-pyrrolidone (NMP). To mitigate the environmental impact of LIB production, aqueous processing has emerged as a state-of-the-art approach, offering faster and more energy-efficient electrode drying.^7^ While various water-soluble biobased polymers such as carboxymethyl cellulose (CMC) and chitosan have been explored as sustainable binders, most of them require additional dispersing agents or chemical modifications to achieve strong interactions with electrode materials and form suitable slurries.^7,9,13^ High-aspect-ratio anisotropic colloids, like nanocellulose isolated from wood, have shown promise due to their excellent network-forming capacity and amphiphilic nature, enabling dispersion of hydrophobic carbon particles without additional surfactants.^14,15^ However, their production often involves extensive and expensive chemical modifications to introduce charged groups, which compromises biodegradability and increases chemical demand.^16,17^

In this context, chitin nanofibers (ChNFs), sourced from marine food waste, are a promising candidate as a binder for graphite electrodes in LIBs. Chitin, structurally similar to cellulose and the load-bearing element in arthropods, possesses a multiscale hierarchical organization with chitin nanofibrils as its fundamental supramolecular unit (Figure 1a).^18,19^ Surface-specific deacetylation of chitin via simple sodium hydroxide treatment selectively generates primary amines on the fiber surface, while the chitin crystalline core is kept intact. This modification promotes lateral disassembly into nanofibrils in acidic conditions (Figure 1b), providing excellent colloidal stability while preserving biodegradability.^20–23^ It is to be noted that the chitin nanofibrils are physiochemically different from chitosan, its extensively deacetylated derivative. While chitosan is a water-soluble polymer defined by its high deacetylation degree (>50% DDA), the ChNF maintains the native, semicrystalline, and high-aspect-ratio nanoscale supramolecular structure and cannot be dissolved in water or any common solvent.

**Figure 1:**
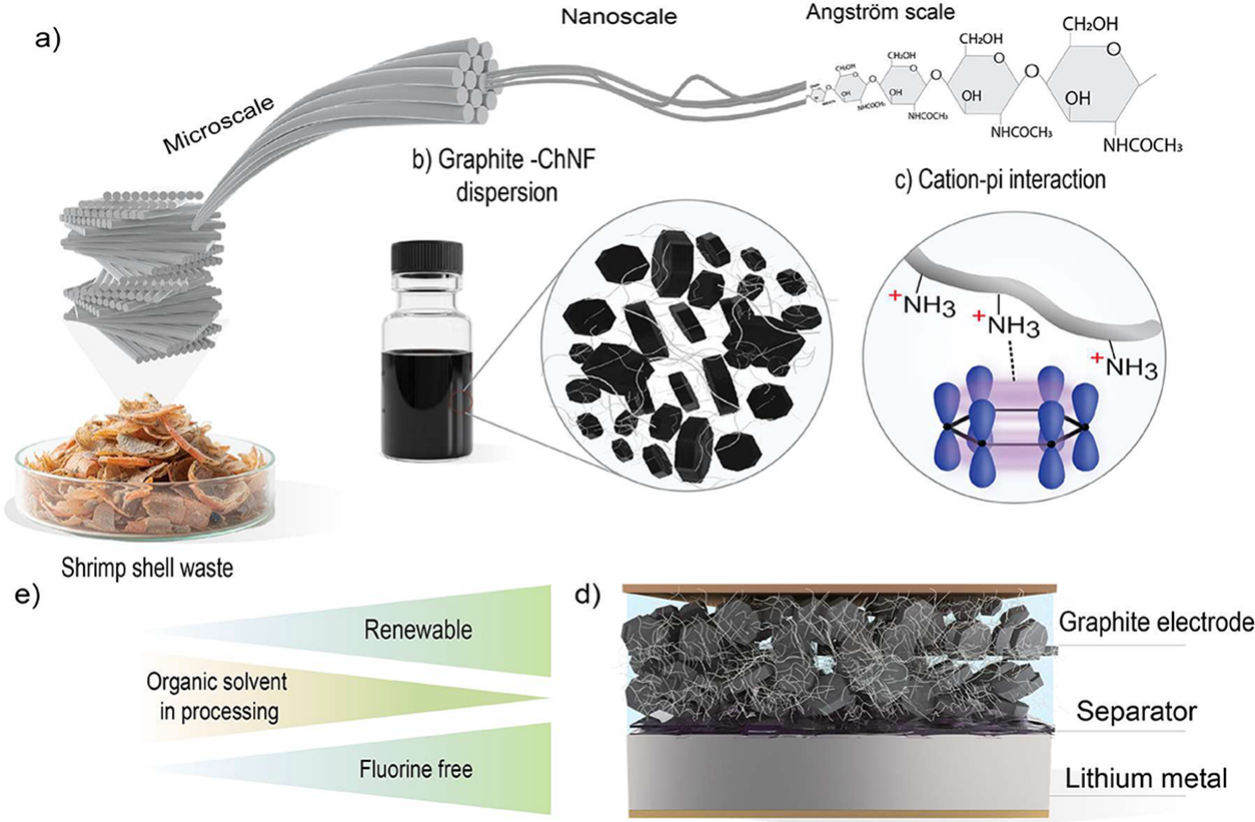
From fishery waste chitin nanofibers to sustainable lithium-ion battery electrodes. (a) Shrimp shell waste is a rich source of chitin fibers, which has a multiscale hierarchical structure. (b) ChNF enabled dispersion of more than 90% of graphite in water. (c) Dispersion of graphite by ChNFs facilitated by a multifaceted stabilizing mechanism: a combination of cation−π interactions, electro-steric stabilization, and entrapment of graphite in the ChNF network. (d) Electrodes fabricated via aqueous processing exhibit excellent electrochemical performance. (e) Renewable and fluorine-free nature of ChNFs offers a sustainable alternative for lithium-ion battery manufacturing.

In this study, we report the unprecedented application of chitin nanofibers as a fluorine-free and sustainable binder, enabling aqueous processing for graphite electrodes in LIBs. With merely 2 wt % ChNFs relative to graphite, the nanofibers enabled the stable dispersion of over 90% graphite in water (Figure 1c). Through systematic rheological analysis and colloidal probe experiments, we elucidated the multifaceted mechanism behind this exceptional dispersibility: the ChNF adsorbs onto graphite via cation–pi interactions (Figure 1d), subsequently providing electro-steric stabilization and ultimately entrapping graphitic particles within the strong gel network formed by the high-aspect-ratio chitin nanofibrils.

Beyond the processing advantage, the electrodes processed via this aqueous approach (Figure 1e) demonstrated enhanced electrochemical performance, including enhanced cycling stability and capacity, compared to conventional counterparts using PVDF binders. To decode the fundamental role of the nanochitin binder in electrochemical performance, we conducted extensive post-mortem analysis, including X-ray photoelectron spectroscopy (XPS) and scanning electron microscopy (SEM) of the electrodes after formation. These investigations, complemented by online electrochemical mass spectrometry (OEMS), provided insights into how the nanochitin binder regulates the formation and characteristics of the solid electrolyte interphase (SEI), revealing its direct impact on battery longevity and efficiency.

## 2 Experimental Section

### 2.1 Binder and Electrode Materials

The nanochitin binder was prepared from chemicals available commercially: chitin from shrimp shell coarse flakes (CAS 1398-61-4, Sigma-Aldrich), sodium hydroxide pellets (CAS 1310-73-2, Titripur Reag., Merck), sodium borohydride (CAS 16940-66-2, Sigma-Aldrich), and aqueous hydrochloric acid (32%, CAS 7647-01-0, VWR chemicals).

The electrode materials, including graphite powder (20 μm), battery-grade lithium metal foil (0.75 mm thickness), polyvinylidene fluoride (PVDF), 1-methyl-2-pyrrolidinone (NMP), and 1 M LiPF_6_ in 1:1 EC:DEC (LP40), were purchased Sigma-Aldrich. Carbon black (Super-P, >99%) was purchased from Alfa Aesar (Thermo Scientific Chemicals). Copper, nickel, and aluminum foil were used as current collectors. All of the materials were used without further purification. LiFePO_4_ (LFP) electrodes of 1 mA h/cm^2^ were used as a combined reference and counter electrode in the mass spectrometric measurements and were supplied by Custom cells GmbH.

### 2.2 Preparation of Chitin Fibers

The chitin nanofibers were produced according to the method described by Arumughan et al.^23^ Briefly, 40 g (dry weight) of chitin fibers was deacetylated in 33% NaOH for 24 h at 90 °C. To minimize depolymerization, 1.2 g of sodium borohydride was added. After the reaction, the product was filtered and thoroughly washed to remove any excess NaOH. The degree of deacetylation (DDA) of the obtained nanofibers was 33.8%, which corresponds to a primary amine content of 1.6 mmol/g.^23^

The deacetylated chitin fibers were dispersed in water at a concentration of 0.3 wt %, and the pH was adjusted to 3 using 0.1 M HCl. The suspension was then homogenized using a high-speed blender for 5 min at 10,000 rpm (T-25Ultra-Turrax Digital Homogenizer, IKA, Germany). The blended suspensions were then subjected to microfluidization using M-110P, Microfluidics Inc., Newton, MA, USA, at a 1500 bar pressure (1 pass). The fluidized suspensions were concentrated using a rotavapor (Büchi R 210, Büchi Labortechnik AG) and stored at 4 °C for further use.

### 2.3 Characterization of Chitin Fibers

#### 2.3.1 AFM

ChNF suspensions with a concentration of 0.005 wt % were deposited on a cleaned silicon substrate using spin coating at 4000 rpm for 1 min. The samples were imaged using a Bruker Multimode 8 AFM in tapping mode. The obtained images were analyzed using Fiber App. The height and length distributions are extracted with the help of a fiber tracking algorithm in the application. The images were processed by using Gwyddion software.

#### 2.3.2 Rheological Measurements

The viscosity was measured using a rheometer (MCR 302, Anton Paar, Germany) equipped with parallel plates (PP25) set at a fixed gap of 0.5 mm. For all measurements, samples were presheared at 100 s^–1^ for 60 s and were left idle for 120 s before starting data acquisition. This protocol ensures homogeneity in the sample. Shear viscosity was monitored across a range of shear rates (0.1–1000 s^–1^) and a strain sweep (0.01–100%) at a fixed frequency of 1.5 Hz. All measurements were performed at 23 °C. To prevent sample evaporation during the tests, silica oil was applied to cover the edges of the parallel plates.

### 2.4 Colloidal Probe Atomic Force Microscopy

#### 2.4.1 Probe Preparation

Tipless cantilevers (HQ:CSC38/tipless/Cr–Au, MikroMasch, Wetzlar, Germany) with spring constants of −0.1 N/m were used. Silicon dioxide (SiO_2_) microparticles (∼30 μm diameter; Polysciences, USA) were attached to the tipless cantilevers using optical adhesive #81 (Norland Products, Cranbury, NJ, USA). The prepared colloidal probes were cured under UV light (365 nm) for 15 min using a UV Cross-linker CL-508 (UVITEC, Cambridge, UK).

Prior to the attachment, the SiO_2_ microparticles were cleaned with piranha solution (3:1 H_2_SO_4_/H_2_O_2_). The colloidal probes underwent additional cleaning using an ozone cleaner (1 h) followed by a 10 min piranha treatment. Cantilever dimensions (width and length) and microparticle (diameter) were determined by using an Olympus BX53 M microscope (Tokyo, Japan). Images of colloidal probes were acquired with scanning electron microscopy (SEM, Phenom Pure G5, Phenom-World, Netherlands).

Colloidal probes were coated with ChNFs (0.1 wt %) via adsorption for 10 min, followed by rinsing with Milli-Q water and drying under nitrogen. Images of the ChNF coating were recorded using a NanoWizard IV XP BioScience AFM instrument (JPK-Bruker, Berlin, Germany) using ScanAsyst Fluid+ probes.

#### 2.4.2 Force Spectroscopy

Force spectroscopy was performed using a NanoWizard IV XP BioScience AFM instrument (JPK-Bruker) in an acoustically isolated system. Calibration was performed prior to each measurement using the thermal noise method to determine the deflection sensitivity and spring constant values.

Measurements were carried out by approaching the colloidal probe to the substrate at a constant velocity of 0.5 μm/s, until a maximum force of 10 nN was reached. Two substrates were tested: (1) a graphene monolayer (theoretical thickness 0.345 nm) deposited on a SiO_2_/Si substrate (Graphenea, San Sebastian, Spain) and (2) a silicon wafer as a control. Measurements were taken at three distinct positions per substrate, mapping a 5 × 5 μm area and recording at least 50 force curves. Experiments were conducted at pH 3, 7, and 10.

The raw force–distance curves, representing the vertical deflection signal as a function of z-piezo displacement, were processed into force–distance curves by using JPK Data Processing software. Further data analysis was conducted using a custom Python script to extract adhesion parameters, including the maximum adhesion force and work of adhesion. Work of adhesion was calculated by integrating the area between the retraction force curve (negative values) and the baseline, while the adhesion force was defined as the minimum point on the retraction curve. In experiments using the colloidal probe technique, forces were normalized by the microparticle radius.

### 2.5 Preparation of Graphite Electrodes

Electrode slurries were prepared with a constant graphite/conductive carbon weight ratio of 95:3. The concentration of chitin fibers varied from 2 to 4% with respect to the weight of the graphite; the amount of water (at pH 3) was kept constant in all formulations. The ChNF suspensions were first homogenized by stirring for 5 min. The graphite and conductive carbon were grounded with a mortar and pestle for 10 min and added to the ChNF dispersion followed by stirring for 1 h at 1500 rpm. The obtained slurry was coated on a copper current collector by using a baker coating applicator forming a coating of 200 μm thickness. Similarly, the 4% PVDF-based slurries were prepared by keeping the same weight percentage with NMP as the solvent. The prepared electrodes were air-dried at room temperature for water-based electrodes and dried using a hot plate at 60 °C for the NMP solvent in air to remove the solvents. After coating, the electrodes were placed in a vacuum oven at 120 °C for 12 h. The dried electrodes were punched into discs of 18 mm diameter, had an active material loading of ∼2.3 mg/cm^2^ and a thickness around 40–50 μm. No calendaring was used before the experiments.

### 2.6 Electrochemical Investigation

All electrodes were investigated in half-cells against lithium metal foil as the counter and reference electrode. The cells were assembled in a single-layer pouch-cell configuration with pouch outer dimensions of 7 × 7 cm. The working electrode and lithium counter electrode (both 18 mm in diameter) were stacked with a Celgard 2320 trilayer separator placed between them. The separator size ensured complete coverage of the electrode area while providing a sufficient margin for vacuum-heat sealing of the pouch material. The electrolyte used was 1 M LiPF_6_ in a 1:1 (v/v) mixture of ethylene carbonate and diethyl carbonate (LP40). Each cell was provided with 150 μL of the electrolyte to ensure thorough wetting of the electrode and separator, corresponding to an electrolyte to capacity (E/C_average)_ ratio of ∼68 μL/mA h. All of the cells were assembled in an argon-filled glovebox (H_2_O < 1 PPM, O_2_ < 1 ppm). Before measurements, the cells were kept at an open-circuit voltage for 5 h to equilibrate the electrolyte throughout the electrodes and separator. The galvanostatic charge and discharge evaluations were conducted within a voltage range of 0–1.5 V using a BioLogic potentiostat. The temperature was maintained at 25 °C as the cells were placed in a Frio cell temperature chamber. Formation cycling was performed at a 0.1 C-rate, followed by rate capability tests at 0.2, 0.5, 1, 2, and back to a 0.1 C-rate, with 30 min rest intervals between each C-rate. Long-term cycling was carried out at a 0.5 C-rate for 100 cycles. The current for each electrode was calculated based on the active mass present in the electrode. Electrochemical impedance spectroscopy (EIS) was performed using a three-electrode EL-cell setup, with frequency ranging from 2.5 MHz to 0.1 Hz and a potential amplitude of 10 mV.

### 2.7 Scanning Electron Microscopy

The morphologies of the pristine and cycled electrodes were analyzed using a Zeiss SigmaVP (Germany) at an acceleration voltage of 3 kV. The cycled cells were disassembled in an argon-filled glovebox. The graphite electrodes were kept under a vacuum oven to remove all of the electrolyte solvents before SEM analysis.

### 2.8 X-ray Photoelectron Spectroscopy

X-ray photoelectron spectroscopy (XPS) analysis was conducted on both pristine and cycled ChNF–Gr and PVDF–Gr electrodes. The cycled cells underwent three formation cycles at a 0.1C rate, followed by full delithiation. The cells were disassembled inside an argon-filled glovebox and placed under a vacuum for 24 h to remove residual electrolyte solvents from the electrode surfaces. The XPS analysis was performed on a Kratos Axis Supra+ using Al-K-alpha radiation. Samples were mounted by using conductive tape and transferred without contact with air from an Ar-filled glovebox. The samples were grounded during the measurement, and the data herein are presented as measured.

### 2.9 Online Electrochemical Mass Spectrometry

Gas analysis was performed using a continuous flow Online Electrochemical Mass Spectrometer (OEMS) in a custom-built setup with a benchtop mass spectrometer from Hiden Analytics, United Kingdom. The mass spectrometer was calibrated with a gas mixture of 2000 ppm of H_2,_ ethylene, CO, CO_2_, and 2000 ppm of O_2_ in argon gas using QGA software. A full spectrum *m*/*z* scan was carried out in Massoft software. Argon gas was used as a carrier gas; all of the ion currents were normalized with ^40^Ar to avoid signal fluctuations. The details about the cell design have been described by Mattinen et al., and the details regarding the updated setup and cell will be coming out in our upcoming publication.^24–26^

For the gas analysis, a LFP||Gr full cell was used to avoid unwanted reactions from lithium metal in classical half-cell experiments. The cell was assembled in the MS cell house by placing a graphite (28 mm diameter) electrode on the bottom followed by the Celgard separator soaked with the LP40 electrolyte and on top the LFP electrode (28 mm diameter) clamped beneath an aluminum current collector with a spring connected to a Gamry potentiostat. For temperature control, the cell house was placed on a temperature plate. To equilibrate the temperature in the cell and ensure uniform distribution of the electrolyte within the pores, the cell was equilibrated for 10 h before the start of the electrochemical measurements. A Gamry potentiostat was used for the measurement. Gas evolution during the formation cycle was analyzed at a 0.1C C-rate, and the gases released from ChNF–Gr were compared to those from PVDF–Gr electrodes.

## 3 Results and Discussion

### 3.1 Chitin Nanofiber Enabled Dispersion of Graphite and Electrode Processing

Achieving a well-dispersed suspension of graphite microparticles in water with suitable rheological properties is crucial for forming a uniform electrode. We have used a graphitic particle with a platelet-like morphology with an average size of 20 μm, and the ChNF has a typical nanofibrilar morphology with an average thickness of 1.5 nm and a length of 705.7 nm that corresponds to an aspect ratio (*a*) of 470.5 (Figure S1). The ChNF contains primary amines as its surface functional group with a density of 1.6 mmol/g that corresponds to a deacetylation degree of 30.46%, which is significantly below the typical threshold (>50% DDA) required to classify the material as chitosan.

The investigation of the dispersing action of ChNF on graphitic particles in water revealed that the addition of as little as 2 wt % ChNFs (relative to graphite) significantly improved the dispersion of graphite in water at pH 3. Furthermore, dispersions containing 3–4 wt % ChNFs demonstrated exceptional long-term stability, remaining homogeneously dispersed for over six months (Figure S2). It is important to note that this stabilization was achieved without the use of additional dispersing agents such as surfactants or styrene butadiene rubber (SBR) as in the case of CMC. To investigate the dispersing mechanism of the ChNF and its impact on the rheological properties of graphitic suspensions, we conducted a comprehensive rheological analysis of both the composite dispersions and the corresponding ChNF suspensions without graphite. The ChNF binder loadings of 2, 3, and 4% (by graphite weight) correspond to ChNF concentrations of 0.66, 0.94, and 1.25 wt % in water (without graphite), respectively. Loadings beyond 4% were avoided because the resulting mixture at high loading formed an excessively thick gel, making electrode processing difficult due to entrapped air.

All ChNF suspensions exhibited shear-thinning behavior, characteristic of anisotropic particle suspensions in semidilute and concentrated regimes (Figure S3).^27,28^ To further understand the nanofibrillar networks formed by ChNFs at different concentrations, we calculated the crowding factor, a dimensionless parameter that measures the extent of fiber packing within a given volume, for each ChNF concentration (Table S1). The computed crowding factors for ChNF suspensions at three concentrations are 713 at 0.66 wt %, 1010 at 0.94 wt %, and 1330 at 1.25 wt %. In all cases, the crowding factor far exceeded the threshold required for a volume-spanning arrested state (*N*_3D_ = *a* = 470). This high crowding factor confirms the formation of a tightly interconnected fibrillar network at all concentrations studied. Figure 2a shows the dynamic viscosities of graphite dispersions at varying ChNF loadings. The results show that all graphitic dispersions exhibited shear-thinning behavior reminiscent of that of pure ChNF networks (Figures 2a and S3). The addition of graphite increased the apparent viscosity compared to ChNF suspensions alone, indicating significant interactions between the graphite particles and ChNF fibers that contribute to an already existing ChNF network (Figure 2b).

**Figure 2:**
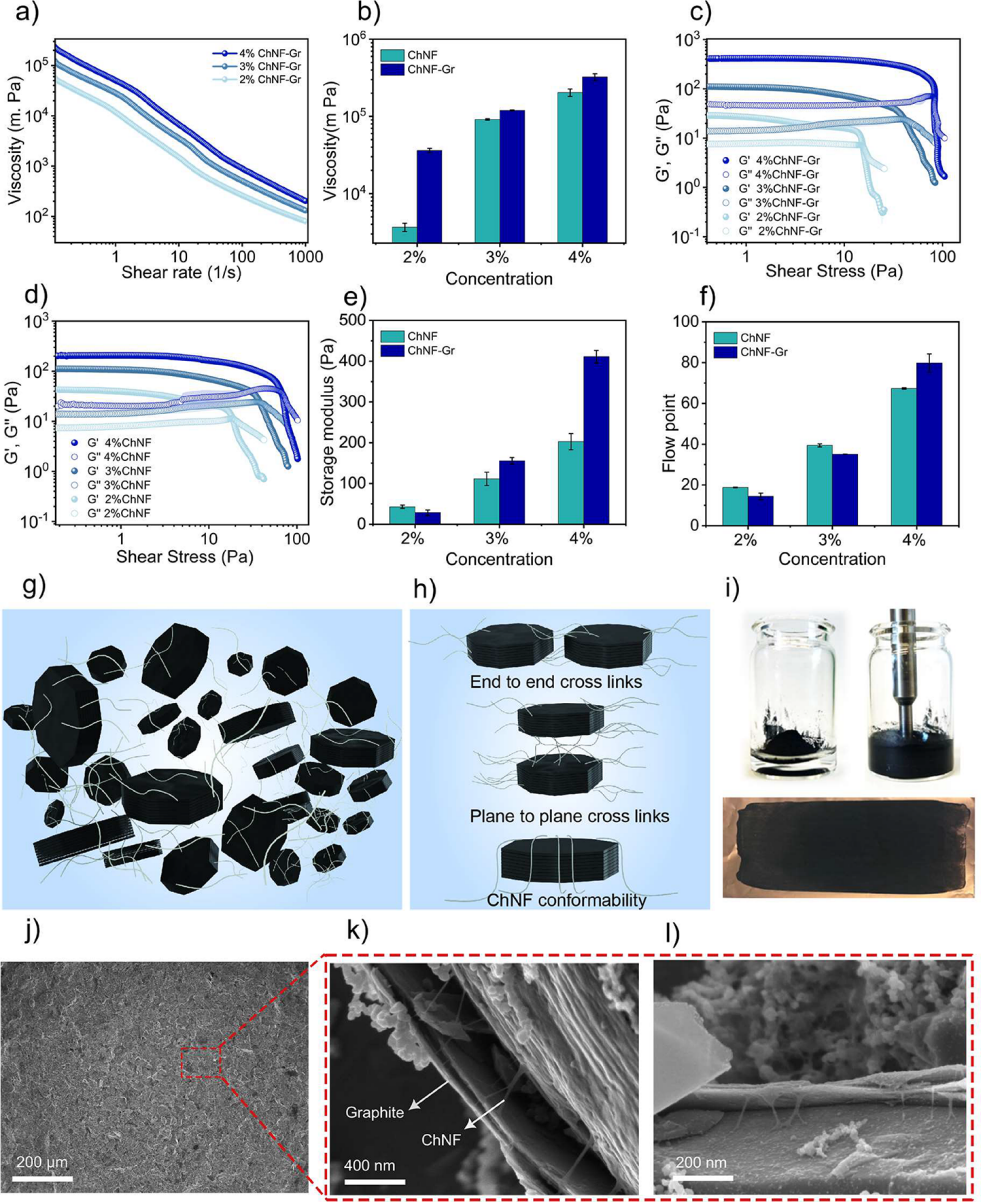
(a) Viscosity of ChNF–graphite composite suspensions as a function of the shear rate. (b) Apparent viscosity of the ChNF–graphite suspensions at different ChNF loadings and that of ChNF suspensions without any graphite. (c) Storage and loss modulus as a function of shear rate for the ChNF–graphite composite suspensions. (d) ChNF suspensions without any graphite. (e) Storage modulus comparison of ChNFs and ChNF–Gr suspensions. (f) Flow point comparison of ChNFs and ChNF–Gr suspensions. (g, h) Illustrations showing the graphite–ChNF network structure and binding modes. (i) Photograph showing slurry preparation (top) and slurry coated on the copper current collector (bottom). (j) SEM image of the pristine ChNF–Gr electrode. (k, l) Magnified view of the ChNF–Gr electrode showing the ChNF holding the graphite.

Amplitude sweep experiments revealed that at low ChNF loading (2–3%) the storage modulus of the ChNF–Gr suspensions was slightly decreased or remained unchanged, suggesting that the apparent viscosity increase observed in these loadings could be due to a hydrodynamic effect (Figure 2c–e). However, at 4% ChNF loading, the storage modulus of ChNF–Gr suspensions showed a significant increase. A similar trend was also visible in the flow point (critical stress), showing that at 4% ChNF loading the addition of graphite particles positively contributed to the fibrillar network (Figure 2f) and confirmed significant graphite–ChNF interactions. Given that the dimensions and the volume fraction of the graphite are significantly larger compared to the ChNF, multiple nanofibrils are needed to connect the graphitic particles either end to end or plane to plane (Figure 2g,h). Due to the flexibility of the nanofibers, they can also conform to the uneven shape of the graphitic particles to yield a well-connected network. The ChNF–Gr suspensions, when processed into electrodes on to the copper plates, consolidated into a very uniform film without any macroscopic cracks (Figure 2i,j). Figure 2k,l shows the plane-to-plane binding of ChNFs with graphitic particles in the dry state.

### 3.2 Molecular Insights into Graphite–ChNF Interactions

To reveal the nature of the graphite–ChNF interaction observed in rheological studies, colloidal probe atomic force microscopy was employed (Figure 3a). It is a powerful technique that enables direct quantification of colloidal forces between surfaces in an aqueous environment.^29–32^ A graphene monolayer (sp^2^-hybridized carbon surface) was used as a graphite substrate model, and silicon oxide (Si/SiO_2_, silicon wafer) was used as a control. The AFM probe was coated with ChNFs via adsorption, resulting in a surface roughness below 50 nm (Figure 3b).

**Figure 3:**
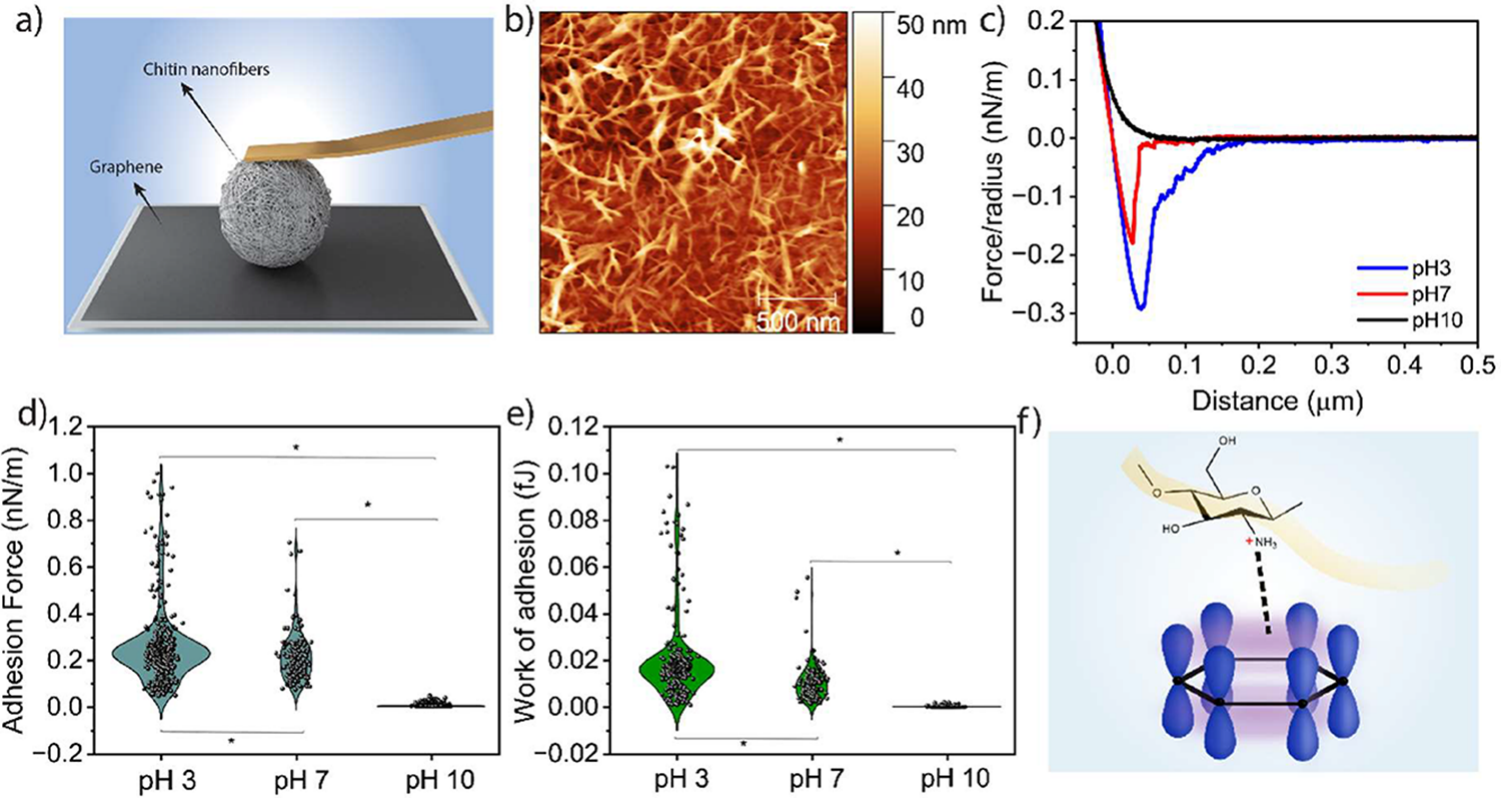
(a) Illustration of the colloidal probe experiment to quantify graphite–chitin interaction. (b) AFM micrograph showing the uniform distribution of ChNFs on the surface of the colloidal probe. (c) Retraction force–distance curves of ChNFs at different pH values. Force normalized by the radius of the probe. The force curves on the approach are not shown. (d, e) Violin plot of work of adhesion and adhesion force at different pH values. Statistical analysis was performed via one-way ANOVA (*p* < 0.05, Tukey’s test). (f) Schematic representation of cation–pi interaction.

Force–distance (F–D) curves were recorded at different pH values (3, 7, and 10) (Figure 3c) to assess the influence of the protonation state of the primary amine on graphite–ChNF interaction. Approached FD curves showed predominantly repulsive forces under all pH conditions (Figure S4). However, distinct adhesion behavior was observed upon retraction. The violin plots in Figure 3d,e depict the distribution of adhesion forces and adhesion energy between ChNFs and the graphene surface (corresponding work of adhesion and average values are given in Table S3). At pH 3, pull-off forces up to (0.255 ± 0.163 nN/m) were observed and the adhesion ranged up to 200 nm. The adhesion gradually decreased as the pH increased. At pH 7, the adhesion force was moderate (0.218 ± 0.106 nN/m), while it dropped dramatically to 0.006 ± 0.006 nN/m at pH 10. This pH-dependent interaction highlights the crucial role of protonation primary amine in governing ChNF–graphene adhesion and suggests the involvement of cation−π interactions between the positively charged chitin surface and the π-electron clouds of the graphitic planes (Figure 3f).^33–35^ This is consistent with a study by Guo et al., which showed that the dispersion of carbon nanotubes in ChNFs increased with a higher degree of deacetylation at acidic pH, further supporting the influence of cation−π interactions.^36^

While the pH-dependent adhesion supports cation–pi interactions, the long-range nature of the adhesion observed (several hundreds of nanometers) exceeded the short-range typical of cation−π interactions (<1 nm). This suggests an additional contribution from a bridging attraction due to the physical extension of ChNF fibrils from the probe before detaching.

To verify that the observed adhesion energy was primarily governed by the sp^2^ carbon lattice of graphene and not by the underlying Si/SiO_2_ substrate, we conducted additional force spectroscopy measurements on bare silicon wafers across a range of pH values (Table S3 and Figure S5). Unlike graphene, the adhesion force and energy on silicon displayed an opposite trend, confirming that the adhesion behavior observed on graphene surfaces was intrinsic to graphene itself and was not influenced by the substrate.

While the colloidal probe experiment demonstrated a strong adhesive interaction between protonated ChNFs and graphite model surfaces, achieving stable dispersion fundamentally necessitates the presence of a repulsive interaction present in the system. We propose a multifaceted stabilization mechanism initiated by the cation–πinteractions. The cation−π interactions facilitate the robust adsorption of protonated ChNFs on graphitic planes. Once adsorbed, the high aspect ratio of ChNFs extends into bulk and provides electro-steric stabilization.^37^ In addition to providing an electro-steric barrier, the high aspect ratio of ChNFs enables strong networks that could effectively entrap larger particles. This physical entrapment can significantly hinder sedimentation and contribute to long-term colloidal stability as established in the case of cellulose nanofibers.^38–40^

### 3.3 Electrochemical Properties of ChNF-Bound Graphite Electrodes

The electrochemical performance of electrodes prepared with 2–4% ChNF–Gr, and a reference electrode 4% PVDF–Gr­(∼2.3 mg/cm^2^), was investigated using galvanostatic charge–discharge cycles in a pouch cell against Li foil, with 1 M LiPF_6_ solution in 1:1 (v/v) ethylene carbonate (EC)/diethylene carbonate (DEC) as the electrolyte. Figure 4a shows the formation cycling of the electrodes at a 0.1 C-rate throughout a potential window of 0–1.5 V, revealing that ChNF binder concentration positively correlates with enhanced cell performance. The initial Coulombic efficiency (CE) was 83.9% for 4% ChNF–Gr and 84.5% for 4% PVDF–Gr, attributed to SEI formation. Both systems stabilized to ∼99% CE by the third cycle, indicating SEI formation completed over subsequent cycles (Table S2). After formation, the 2, 3, and 4% ChNF–Gr electrodes delivered delithiation capacities of 292, 289, and 370 mA h g^–1^, respectively, while the 4% PVDF–Gr reference reached 350 mA h g^–1^. The lower initial specific capacity observed for electrodes with a lower ChNF percentage is due to an insufficient binder content to form a fully interconnected electrode network. As indicated by the rheological measurements, at 4% ChNF loading, a stronger network formation with graphite is promoted, improving electrode integrity and electronic connectivity. To compare the performance of the ChNF binder with the existing water-based binders, a literature survey was carried out and is summarized in Table S4. The aqueous-based binders show typical specific capacities from 290 to 382 mA h/g. Among them, sodium alginate and chitosan exhibited higher initial capacities with higher binder contents.^41^ However, at a binder content of 4%, the ChNF binder is superior to that of the reported alternatives. Additionally, the performance of our PVDF reference electrode aligns well with literature values, ensuring a fair and reliable basis for comparison.

**Figure 4:**
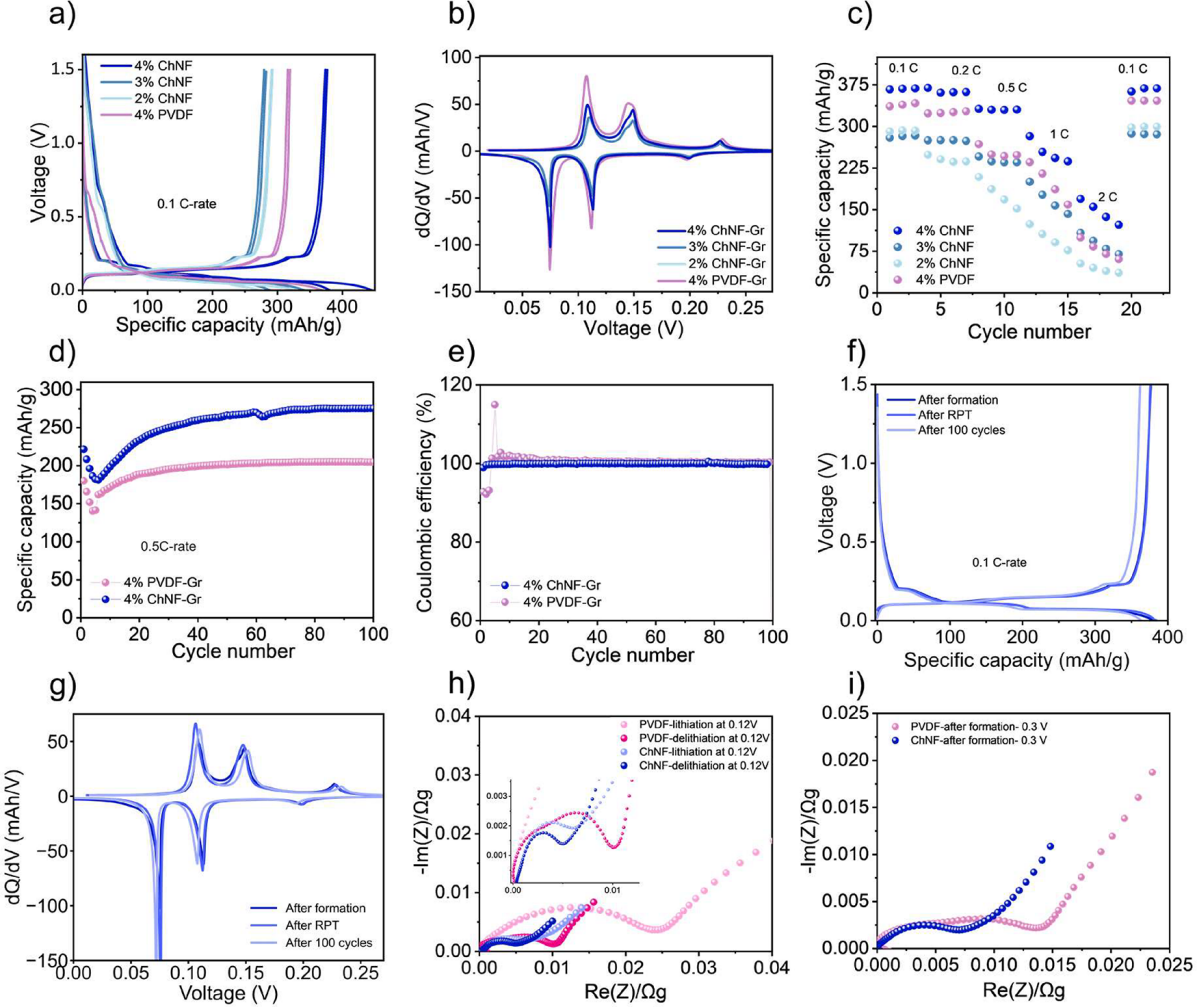
Characterization of the electrochemical properties of ChNF–Gr and PVDF–Gr electrodes. (a) Charge and discharge voltage profiles of 2, 3, 4% ChNF–Gr, and 4% PVDF–Gr electrodes during 3 formation cycles at a 0.1 C-rate. (b) d*Q*/d*V* curves of the 3rd formation cycle. (c) Rate performance with 0.1–2C-rates. (d) Long-term cycling of 4% ChNF–Gr and 4% PVDF–Gr electrodes at a 0.5 C-rate. (e) Coulombic efficiencies of the long-term cycling. (f) Capacity comparison of 4% ChNF–Gr electrodes after formation, RPT, and long-term cycling at a 0.1 C-rate. (g) Corresponding d*Q*/d*V* curve. (h) EIS of 4% ChNF–Gr and 4% PVDF–Gr electrodes during the first lithiation and delithiation (inset shows the enlarged image). (i) EIS comparison after formation.

To further elucidate the electrochemical behavior, incremental capacity analysis (d*Q*/d*V* curves) was performed to identify voltage plateaus corresponding to lithiation and delithiation processes.^42,43^ Figure 4b illustrates the d*Q*–d*V* curves of the corresponding formation cycles. The d*Q*/d*V* profiles revealed oxidation and reduction peaks for ChNF–Gr electrodes similar to PVDF-based systems, confirming that the ChNF binders preserve electrochemical stability without compromising the graphite’s performance. Rate capability tests (0.1–2C) showed that ChNF–Gr electrodes maintained stable capacity up to 0.5C, with an expected decline at higher rates (Figure 4c). The 4% ChNF–Gr electrode outperformed others, and all variants exhibited excellent capacity recovery upon returning to 0.1C.

Cycling tests were conducted on the 4% ChNF–Gr and 4% PVDF–Gr electrodes over 100 cycles at a 0.5C rate (Figure 4d). The ChNF–Gr electrode delivered a specific capacity of approximately 270 mA h g^–1^, significantly higher than that of the PVDF–Gr counterpart (∼200 mA h g^–1^). In both cells, a capacity dip followed by an increasing trend is observed, which might be attributed to the interlayer changes on the graphite electrodes during high depth-of-discharge conditions, facilitating Li^+^ ion diffusion.^44^ Both electrodes exhibited stable cycling performance over 100 cycles, and the Coulombic efficiency of both cells remained above 99% throughout the test (Figure 4e). The superior performance of the ChNF–Gr electrode is presumably due to the formation of a stable SEI.^45^ Even under prolonged cycling, the SEI remains stable to maintain a consistent electrode–electrolyte interface.^46^ After a rate performance test and long-term cycling, the ChNF–Gr electrode retained a capacity of 362 mA h g^–1^ (Figure 4f) with minimal curvature changes in d*Q*/d*V* profiles (Figure 4g), confirming the stability of the lithiation equilibria between different electrochemically active phases. It should be noted that the electrochemical performance of the ChNF–Gr cells has proven to be reproducible across multiple experiments.

The electrochemical impedance spectra (EIS) of the 4% ChNF–Gr and 4% PVDF–Gr electrodes were recorded with a three-electrode EL cell. The initial lithiation and delithiation cycles were monitored at 1 h intervals, with the corresponding impedance evolution plotted in Figure S6. Figure 4h specifically highlights the EIS spectra obtained at 0.12 V (approximately 25% SOC) during the first cycle. The high-frequency resistances of 0.07 Ω for ChNF–Gr and 0.2 Ω for PVDF–Gr are associated with the electrolyte resistance. This contribution is excluded from the EIS spectra because it is independent of the active mass and therefore cannot be properly normalized by mass. The Nyquist plot, observed during the stage transition in both the electrodes, showed flattened semicircles and a Warburg tail in the low-frequency region.^47,48^ Generally, the impedance observed in the first lithiation is associated with the formation of the SEI layer and is higher compared to the delithiation. Comparing the two electrodes, the ChNF–Gr electrode exhibited a smaller semicircle height and width than the PVDF–Gr electrode. The maximum semicircle height and width are more than three times larger at lithiation for PVDF–Gr and double at the delithiation, confirming lower interfacial resistance and improved charge-transfer kinetics in ChNF–Gr. The evolution of EIS after three formation cycles was also measured at 0.3 V (50% SOC), as shown in Figure 4i. The semicircle width is still double for the PVDF–Gr but the heights are relatively similar. The more depressed semicircle for the PVDF–Gr could be due to a combination of charge-transfer processes but is likely an effect of a larger current distribution in this electrode.^49^ Moreover, the Warburg resistance is smaller for the ChNF, indicating that the diffusion properties are also affected by the choice of the binder. The lower impedance of the ChNF–Gr cells compared to the PVDF–Gr cells aligns well with the higher electrochemical performance of the ChNF–Gr cell.

### 3.4 Binder-Regulated SEI Formation on Nanochitin–Graphite Electrodes

Scanning electron microscopy was used to investigate the microscopic structure of the 4% ChNF–Gr and 4% PVDF–Gr electrodes after formation cycling. The SEM images of PVDF–Gr and ChNF–Gr electrodes (Figure 5a,c) show dense and uniformly packed electrodes with no cracks. The magnified view of the PVDF–Gr and ChNF–Gr electrodes in Figure 5b and Figure 5d shows significant differences in the surface structure, also different from the pristine sample (Figure S7a,b). In PVDF–Gr electrodes, the surface is covered by a thin uniform layer.^50^ In contrast, the ChNF–Gr electrodes exhibit a thick layer deposition along with additional aggregations on the surface. The statistical significance of this observation has been verified (see Figure S8a–c).^51^ This difference in morphological features indicates the influence of SEI formation on binders.

**Figure 5:**
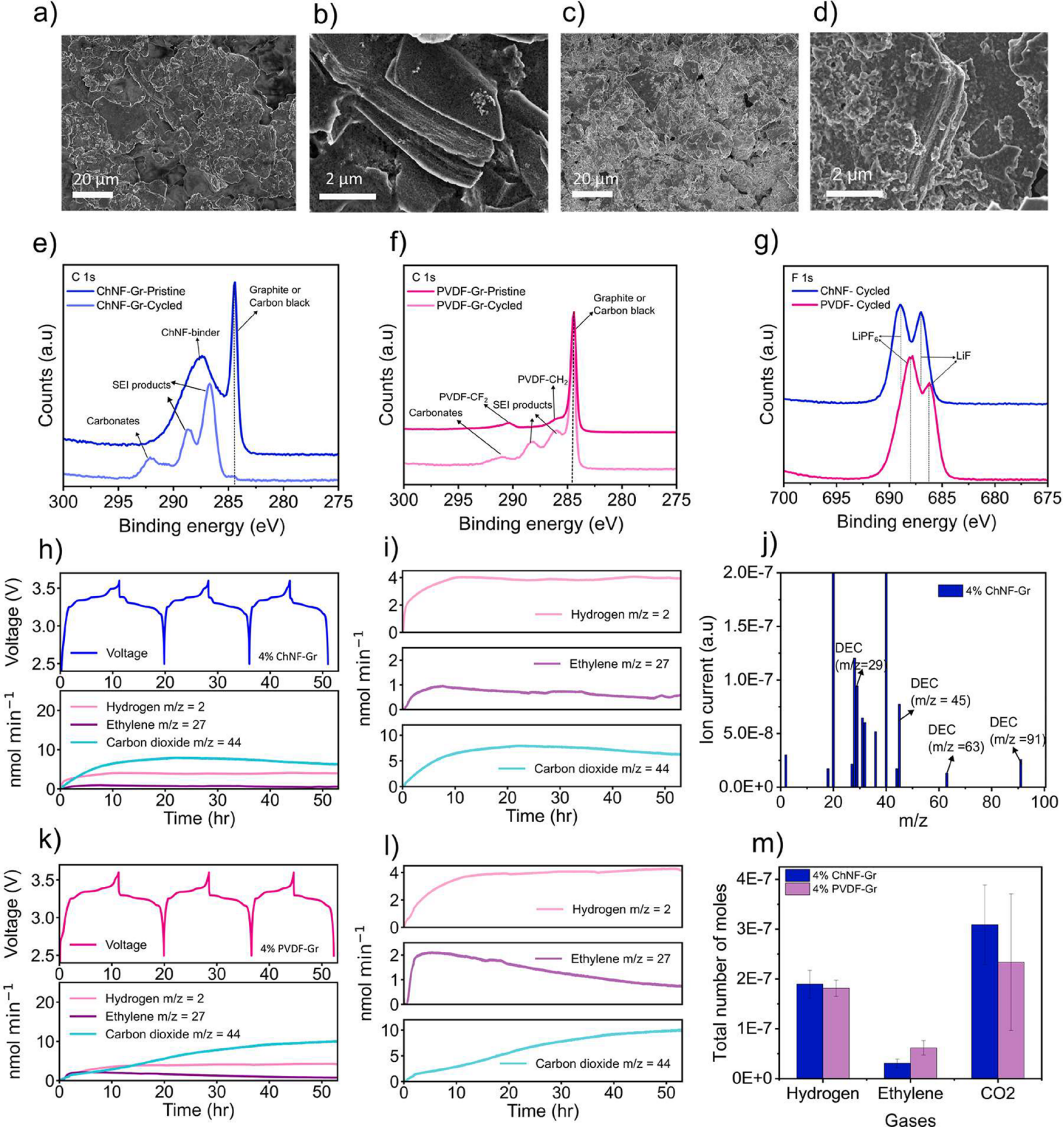
SEM image of the 4% PVDF–Gr electrode after formation at (a) 1k and (b) 10k magnifications. SEM image of the 4% ChNF–Gr electrode after formation at (c) 1k and (d) 10k magnifications. XPS results of graphite electrodes: (e) C 1s spectra of pristine and cycled ChNF–Gr electrodes; (f) C 1s spectra of PVDF–Gr pristine and cycled electrodes; and (g) F 1s spectra of cycled ChNF–Gr and PVDF–Gr F 1s electrodes. OEMS measurement of first three formation cycles of ChNF–Gr and PVDF–Gr electrodes. (h) Voltage profile of 4% ChNF–Gr electrodes during formation at a 0.1 C-rate on the top and corresponding gas evolution (bottom) for H_2_ (rose), ethylene (purple), and carbon dioxide (blue). (i) Enlarged individual gas trend curves from (h); (j) full scan spectra of ChNF–Gr cell after the formation; (k) voltage profile of the 4% PVDF–Gr electrode (top) and corresponding gas evolution (bottom), (l) individual gas features from (k); and (m) total gas evolved in the ChNF–Gr (blue) and PVDF–Gr (pink) electrodes.

Further surface characterization was carried out using X-ray photo electron spectroscopy (XPS) on both the pristine and the cycled samples. Figure 5e,f shows the C 1s spectra of ChNF–Gr and PVDF–Gr. In the pristine state, both spectra show a signal from the graphite or carbon black at about 284.5 eV. The characteristic peaks of the CF_2_ and CH_2_ peaks from the PVDF binder and a broad feature centered at around 288 eV for the ChNF binder are observed. After cycling, peaks from SEI products and carbonates emerge, while the peaks stemming from the binders decrease in relative intensity, indicating that surface layers are being formed on the electrode. The graphite/carbon black signal can still be observed in the PVDF–Gr electrodes, while it is almost completely diminished in the ChNF electrodes. This would indicate that a thicker surface layer is formed in the ChNF electrode compared to the PVDF–Gr in agreement with the SEM observation.^45^ The F 1s spectra (Figure 5g) reveal the contributions from the LiPF_6_ salt and its decomposition products, primarily LiF. Even though both electrodes show similar features, the spectra of the cycled ChNF–Gr are shifted to a higher voltage of 1 eV compared to those of PVDF–Gr.^52^ Similar trends are also observed in the O 1s spectra (Figure S9). This could possibly be the result of a different electrochemical potential difference at the graphite SEI interface in the ChNF–Gr, possibly related to the formation of a thicker SEI layer for this system or variations in the surface depositions in the ChNF–Gr system.

To elucidate the mechanisms of the SEI formation, gaseous species generated during the formation were analyzed using OEMS for 4% ChNF–Gr and 4% PVDF–Gr electrodes vs LiFePO_4_. Figure 5h,k shows the gas evolution rate plotted against the cell voltage, and Figure 5i,l shows the detailed view of gases for ChNF–Gr and PVDF–Gr cells, respectively. The evolution rates of three gases, hydrogen (*m*/*z* = 2), ethylene (*m*/*z* = 27), and carbon dioxide (*m*/*z* = 44), were monitored and quantified.

The evolution of H_2_ gas was similar in both cells (Figure 5i,l top). Both the cells produced H_2_ gas at the rate of ∼4 nmols/min at the maximum cut off voltage, stabilizing during discharge. A straight trend was repeated throughout the cycling process. The results show that H_2_ was the first gas to evolve at the beginning of the gas cycling and is predominantly produced via the reduction of residual water in the electrolyte at the graphite surface.^53,54^ Additionally, the Li^+^ can electrochemically react with water to form LiOH and H_2_, with the further reduction of LiOH also leads to the formation of H_2_. Furthermore, PF_6_^–^ hydrolysis in the presence of trace water produces HF, which can react with Li^+^ to form H_2_ (see Figure S10 and eqs 1–5).^55^

Ethylene evolution is a characteristic of EC reduction during the SEI formation on graphite electrodes. The ethylene evolution profiles in both cells increased during the first charging step, gradually decreased in subsequent cycles, but differed significantly in quantity (Figure 5i,l middle). The maximum ethylene production was ∼1 nmol/min in the ChNF–Gr cell, whereas it was nearly double ∼2 nmol/min in the PVDF–Gr cell. For the ChNF–Gr cells, the higher H_2_ evolution in the initial period of cycling suggests the deposition of inorganic products such as LiOH, Li_2_O, and LiF on the surface of the graphite. This deposition could cover the graphite surfaces, limiting EC reduction and thus the ethylene production. This also suggests a reduced formation of solid deposits such as lithium diethylene carbonate (LEDC) or lithium carbonate (Li_2_CO_3_) (Figure S10 and eqs 6 and 7).^55–57^ On the other hand, in the PVDF–Gr cell, the lower H_2_ evolution in the initial cycling and higher ethylene production indicate higher deposition of LEDC and Li_2_CO_3_, reflecting the binder-dependent SEI formation.

The CO_2_ evolution began during the open-circuit potential period in both cells due to chemical degradation of EC in the presence of water already present in the electrolyte (Figure S9: eq 8).^55,58^ CO_2_ showed distinct behaviors between the two electrodes (Figure 5i and l bottom). In ChNF–Gr, CO_2_ peaked at 7.8 nmol/min during the first cycle and declined thereafter, while PVDF–Gr showed a continuous increase to 9.9 nmol/min until the end of the cycle. In ChNF–Gr, the H_2_ and CO_2_ evolutions are correlated_,_ suggesting that the hydroxides coming from water reduction or alkoxides can initiate the autocatalytic hydrolysis of EC and generate CO_2_ and the deposition of (poly)­ethylene glycol on the surface of the graphite (Figure S9: eq 9).^58,59^ However, in the PVDF–Gr cell, the dominant reaction presumably is the decomposition of the LEDC to lithium ethyl methyl carbonate (LEMC) and CO_2_ in the presence of H_2_O or HF (Figure S9: eqs 10–11) consistent with the higher ethylene output.^55^

To understand the additional gaseous species formed during cycling, a full mass scan from 0 to 100 *m*/*z* was recorded. Apart from the three gases we have investigated, the scan also revealed argon gas peaks at *m*/*z* 20 (doubly charged), 36 (isotope), and 40, water at *m*/*z* = 18; and DEC solvent fragments at *m*/*z* = 29, 31, 45, 63, and 91. These were also present in both the cells, confirming the absence of unexpected byproducts (Figures 5j and S10). The cumulative gas evolution showed similar H_2_ and CO_2_ levels in both the cells, with a significantly higher ethylene release in PVDF–Gr. (Figure 5m). The larger variation in CO_2_ measurements compared to H_2_ and C_2_H_4_ is attributed to the fact that the latter gases originate mainly from specific electrochemical reactions, while the CO_2_ evolution originates from both electrochemical reactions and chemical decomposition processes of the electrolyte. In addition, CO_2_ is known to also be adsorbed and consumed in the cell, and therefore, its concentration is not only dependent on the electrochemical protocol but also on other factors such as time. This suggests that EC reduction is predominant with the deposition of LEDC followed by its degradation, while in the ChNF–Gr electrode along with the LEDC, (poly) ethylene glycol was the major deposit according to the OEMS results. Given the hydroxyl-rich structure of ChNFs, we propose a reaction pathway involving PF_5_ binding to hydroxyl groups, forming lithium fluorophosphates, as reported for CMC and PAA binders (Figure S9: eq 12).^60,61^ It is evident from the OEMS studies that the mechanism for the SEI formation differs significantly depending on the electrode binder, resulting in a distinct morphology and composition of the SEI layer on PVDF- and ChNF-based electrodes (Figure 6). Specifically, the ChNF–Gr electrode forms an SEI that is thicker than that on the PVDF electrode, yet it exhibits a surprisingly low charge-transfer resistance. This low resistance, despite the thickness, is likely due to favorable compositional differences or porosity. Overall, this unique SEI, which is thicker but allows for faster ion movement, is highly beneficial because it effectively limits continuous electrolyte decomposition by preventing the direct contact between the electrolyte and the graphite surface, thus contributing to the superior electrochemical performance of the ChNF–Gr electrode.

**Figure 6:**
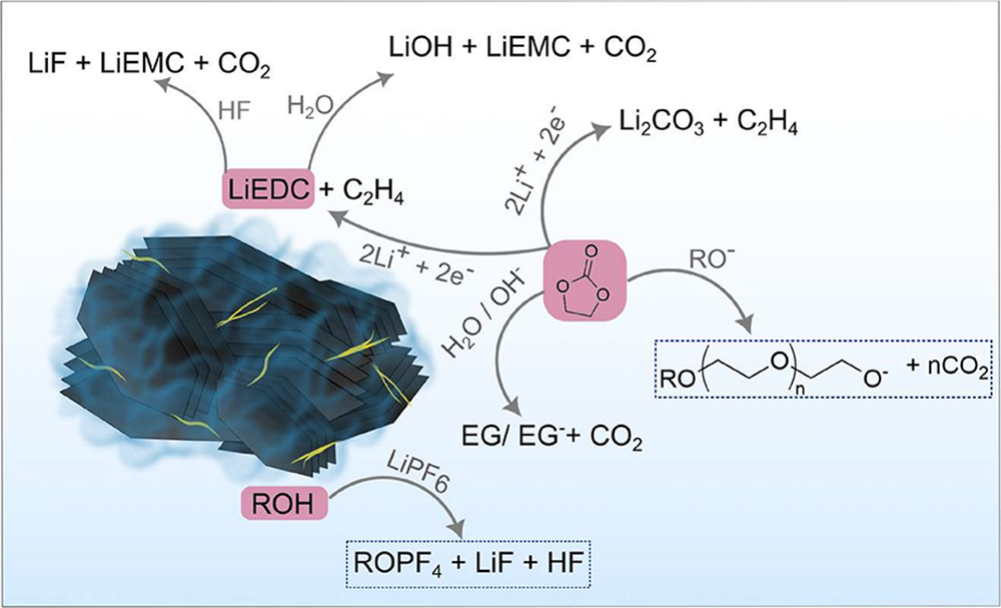
SEI formation mechanism on the graphite electrode (the highlighted reactions are taking place specifically in the ChNF–Gr cells).

## 4 Conclusions

This study presents marine-waste-derived chitin nanofibers as an alternate fluorine-free binder for LIB anodes. We demonstrated that ChNFs enable the stable dispersion of over 90 wt % graphite in water, without the need for chemical modification or additional additives, thus facilitating environmentally benign, water-based electrode fabrication. CPM revealed pH-dependent adhesion of protonated ChNFs and graphitic surfaces likely stemming from a cation–π interaction. The pH-dependent adhesion facilitates an exceptional dispersing action of ChNFs through a multifaceted mechanism. Once absorbed onto graphite, ChNFs provide electro-steric stabilization by extending into the aqueous phase. In addition to that, the strong network formed by ChNFs could physically entrap larger particles, significantly improving the long-term stability of ChNF–graphite suspensions.

The electrochemical characterization revealed that the 4 wt % ChNF formulation demonstrates enhanced capacity retention over 100 cycles, surpassing the performance of the conventional PVDF binder at similar loadings. This improved performance is attributed to the binder’s positive influence on the solid electrolyte interphase. Through a combination of morphological analysis via SEM, surface composition analysis by XPS, and insights from online electrochemical mass spectrometry, we decoded how the nanochitin binder actively regulates SEI formation pathways. Notably, ChNF–graphite electrodes promote the formation of a uniform and stable SEI layer that effectively passivates the graphite surface, minimizing continuous electrolyte decomposition. The observed presence of SEI agglomerates, likely stemming from reactions involving ChNF hydroxyl groups, highlights the unique and beneficial interfacial chemistry afforded by the nanochitin binder. Collectively, this work establishes ChNFs as a high-performance and sustainable binder for LIB anodes, offering a viable and greener alternative to fluorinated polymers and is compatible with other biobased binders such as CMC or chitosan without requiring additional dispersing agents or chemical modifications to achieve strong interactions with electrode materials. These findings not only open new avenues for the design of biobased components in next-generation energy storage systems but also provide critical insights into binder-dependent SEI formation pathways for optimizing battery longevity and efficiency. The promise of chitin as a binder for positive electrodes will be evaluated in a separate study.

## Supplementary Material



## Notes

AFM image of ChNFs with length and height distributions; the photograph of ChNF–Gr dispersion; viscosity of ChNF suspensions as a function of the shear rate; critical overlap concentration and crowding factor calculations; the approach and retraction force–distance curves for ChNFs and graphene interactions at different pH values; average values and the violine plot of work of adhesion and adhesion force for graphene and silicon wafer at different pH values; EIS spectra of ChNF–Gr during de/lithiation in 1 h intervals of time during the first formation; the SEM image of pristine and cycled electrodes; XPS spectra of oxygen 1s of cycled ChNF–Gr and PVDF–Gr electrodes; gas evolution reaction mechanisms associated with SEI formation in graphite electrodes; specific capacities of graphite electrodes with the PVDF and the reported water-based binders; and full scan spectra of the PVDF–Gr cell after the formation (PDF)
