# Supplementary material for: Nanochitin as Binder in Li-Ion Battery Anodes Enabling Aqueous Processing and Superior Solid Electrolyte Interphase
Source: ACS Appl Mater Interfaces. 2026 Apr 18;18(16):23181–92. doi: 10.1021/acsami.6c01089 (PMC13133777; doi:10.1021/acsami.6c01089)
Supplement: Supplementary file 1 [file am6c01089_si_001.pdf]

Supporting information

Nanochitin as Binder in Li-ion Battery Anodes Enabling Aqueous Processing and Superior Solid Electrolyte Interphase

Amritha P. Sandra[a*], Vishnu Arumughan[b*], Roberta Teixeira Polez[b], Fredrik Lindgren[c], Maria Hahlin[c,d], Han Tao[b], Monika Österberg[b], Eero Kontturi[b], Rakel Wreland Lindström[a*]

[a] *Department of Chemical Engineering, KTH Royal Institute of Technology, SE-100 44 Stockholm, Sweden*

[b] *Department of Bioproducts and Biosystems, School of Chemical Engineering, Aalto University, Espoo, FI-0076 Aalto, Finland*

[c] *Department of Chemistry−Ångström Laboratory, Uppsala University, Box 538, SE-751 21 Uppsala, Sweden*

[d] *Department of Physics and Astronomy, Uppsala University, Uppsala 751 20, Sweden*

Results

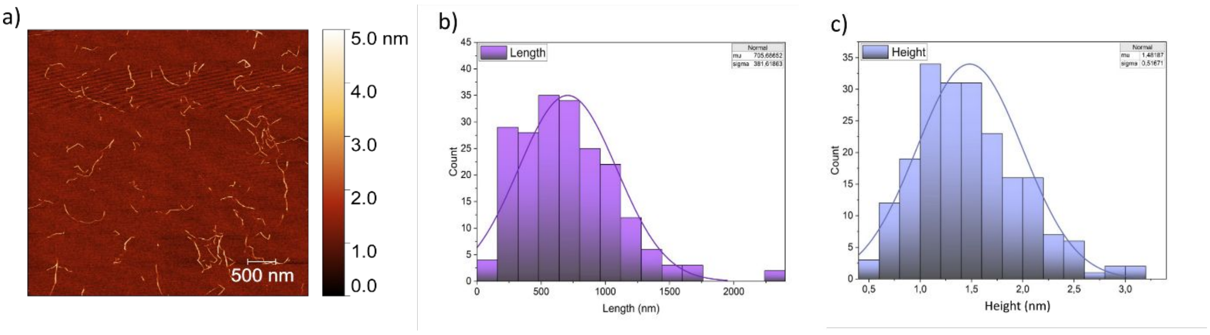

Figure S1: a) AFM image of ChNF suspension deposited on SiO_2 wafer. b) length distribution and c) height distribution.

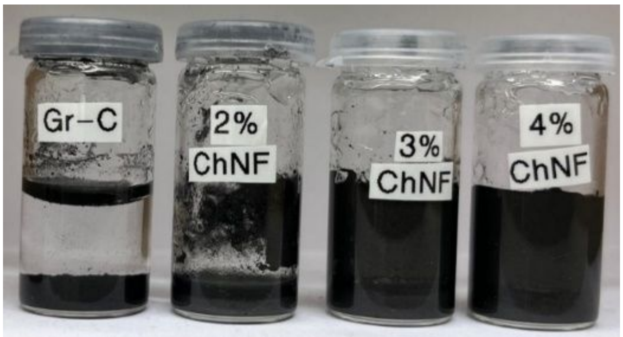

Figure S2: Picture of ChNF-Gr dispersion stored at room temperature for 6 months.

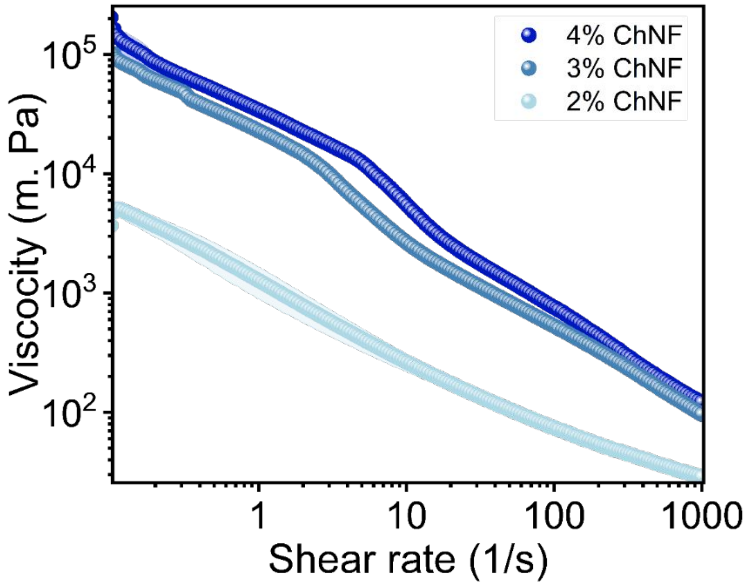

Figure S3: The viscosity of ChNF suspensions as a function of shear rate.

Critical overlap concentration and crowding factor calculations

The high aspect ratio of anisotropic ChNF facilitates the formation of robust networks. These networks can be mathematically described by using concept of overlap concentration and crowding factor. In this calculation the ChNF was assumed to have a geometry of nanorods which has double layer repulsion. The critical overlap concentration (C_{OL}) and crowding factor (N) are computed as follows.

Critical volumetric overlap concentration ($C_{v,OL}$) = $\frac{3d^2}{2L^2}$

Where d is average thickness and L is average length obtained from AFM analysis.

Critical weight-based overlap concentration ($C_{w,OL}$) = $\frac{1.37\ C_{V,OL}}{1.37\ C_{V,OL}+(1-\ C_{V,OL}\ )*1}$

Where the density of chitin (ρ_{chitin}) is 1.37 g/mL and density of water (ρ_{water}) us 1.0 g/ml

The crowding factor (N) is given by

$$N=\frac{2}{3}C_V\frac{L^2}{d^2}$$

Table S1: Crowding factor calculation.

Sample	Diameter	Length	$C_{v.OL}$	$C_{w.OL}$
ChNF	1.5	705.7	6.77 E-06	9.28 E-06

Sample	Crowding factor (N)
0.66 %	713
0.94 %	1010
1.25 %	1330

Table S2: Columbic efficiencies of the formation cycles.

Electrode type	Columbic efficiency of 1st cycle (%)	Columbic efficiency of 3rd cycle (%)	Standard deviation (±)
**4%ChNF-Gr**	83.9	99.0	2.57
**3%ChNF-Gr**	81.9	98.8	2.32
**2%ChNF-Gr**	83.5	98.9	9.9
**4%PVDF-Gr**	84.5	98.7	17

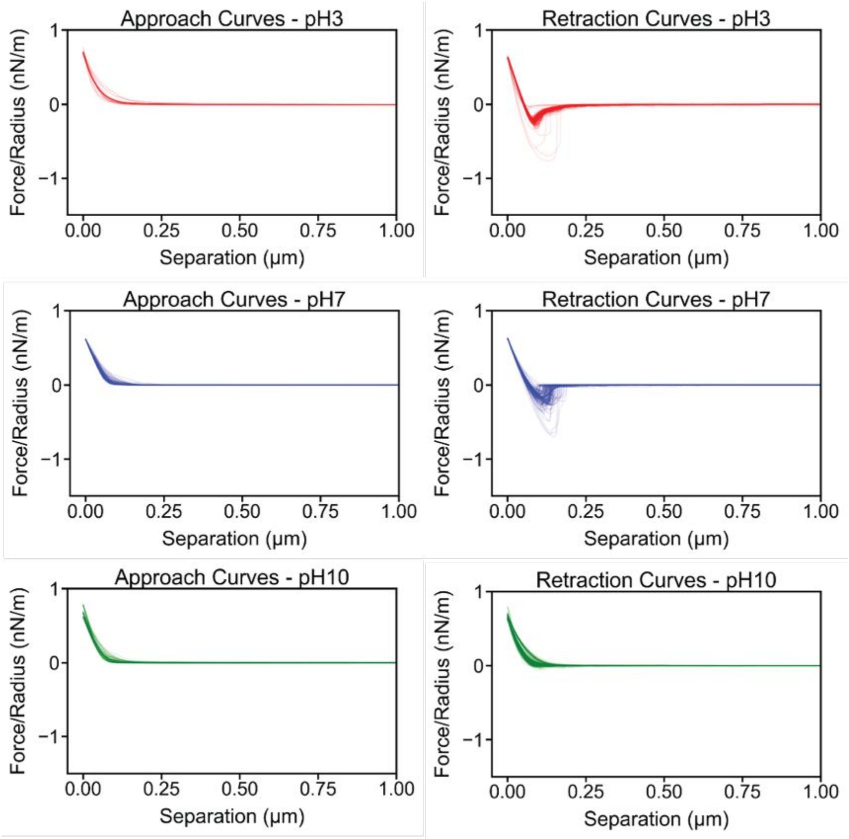

Figure S4: Approach and retraction force-distance curves for ChNF and graphene interactions at pHs 3, 7 and 10. Force normalized by the radius of the probe.

Table S3: Average values of work of adhesion and adhesion force for graphene and silicon wafer at different pHs. Statistical analysis was performed via one-way ANOVA ($p < 0.05$, Tukey's test). Different letters indicate significant statistical differences

**Substrate**	**pH**	**Work of adhesion (fJ)**	**Adhesion force (nN/m)**
Graphene	3	0.019 ± 0.017 ^{A}	0.255 ± 0.163 ^{E}
	7	0.010 ± 0.008 ^{B}	0.218 ± 0.106 ^{E,F}
	10	0.00004 ± 0.0002 ^{D}	0.006 ± 0.006 ^{J}
Silicon	3	0.008 ± 0.010 ^{B,C}	0.157 ± 0.130 ^{G}
	7	0.005 ± 0.007 ^{C}	0.062 ± 0.069 ^{H}

10	0.016 ± 0.029 ^{A}	0.203 ± 0.245 ^{F}

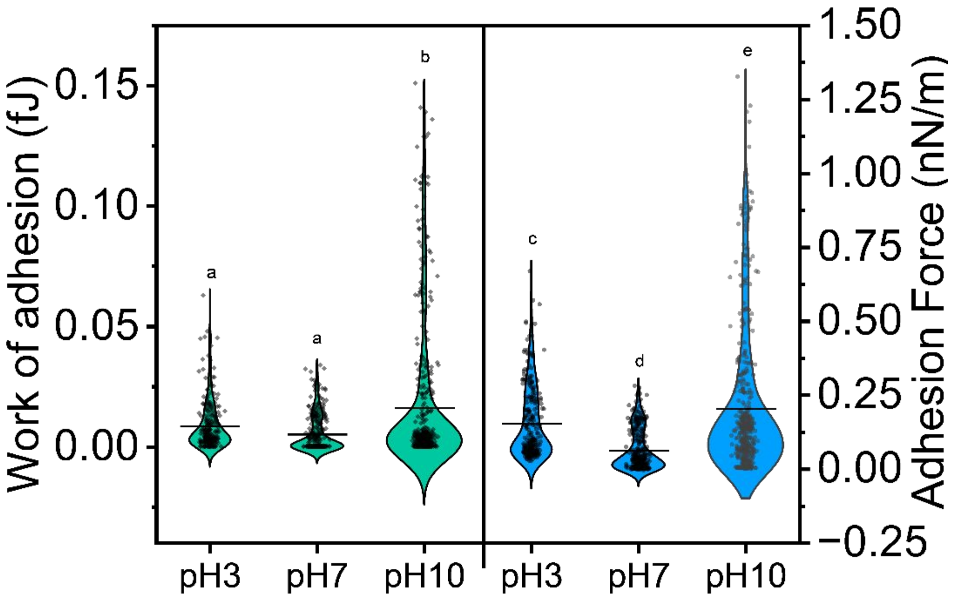

.

Figure S5: Violin plot of work of adhesion and adhesion force between ChNF and silicon wafer at different pHs. Statistical analysis was performed via one-way ANOVA ($p < 0.05$, Tukey's test). Different letters indicate significant statistical differences.

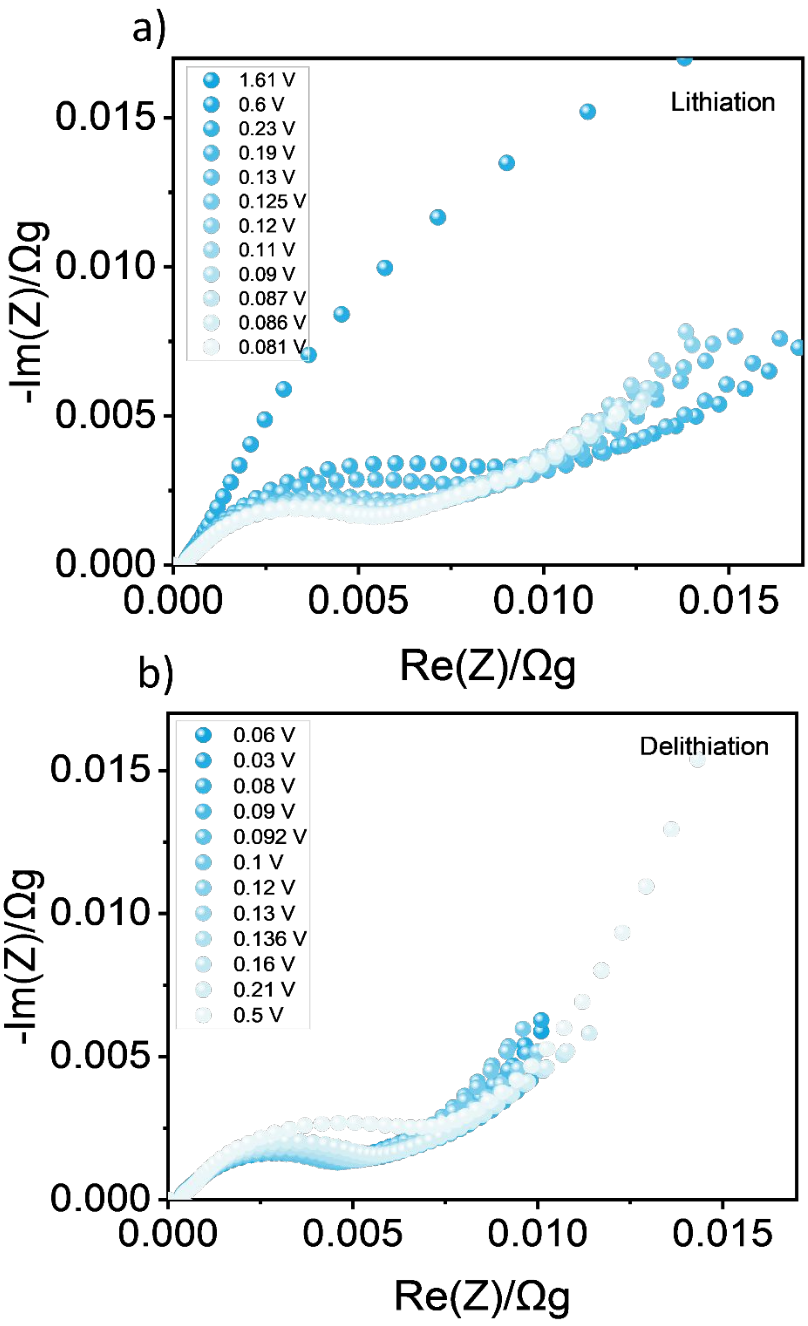

Figure S6: a) EIS spectra of ChNF-Gr during lithiation (b) during delithiation in one hour interval of time during the first formation at 0.1 C-rate.

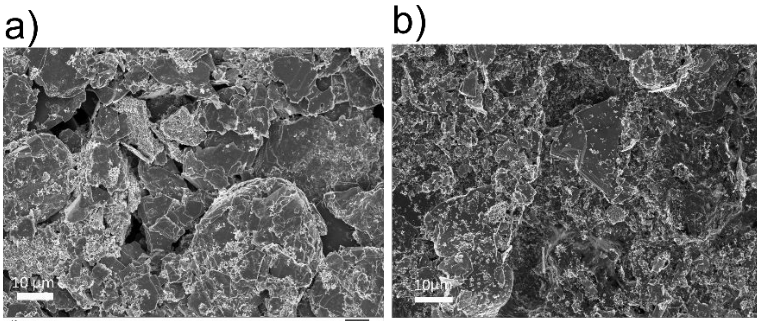

Figure S7: SEM image of pristine electrodes: a) PVDF-Gr cell a, b) ChNF-Gr cell.

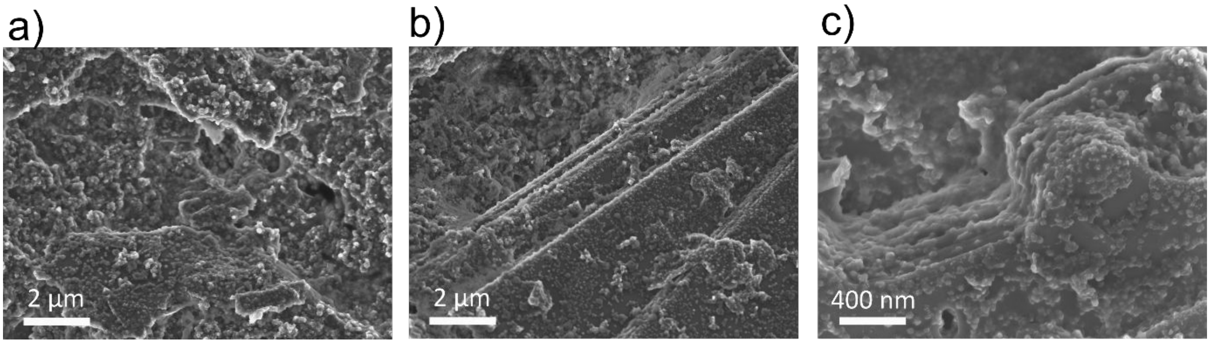

Figure S8: SEM image of ChNF-Gr cell (a-c) after formation.

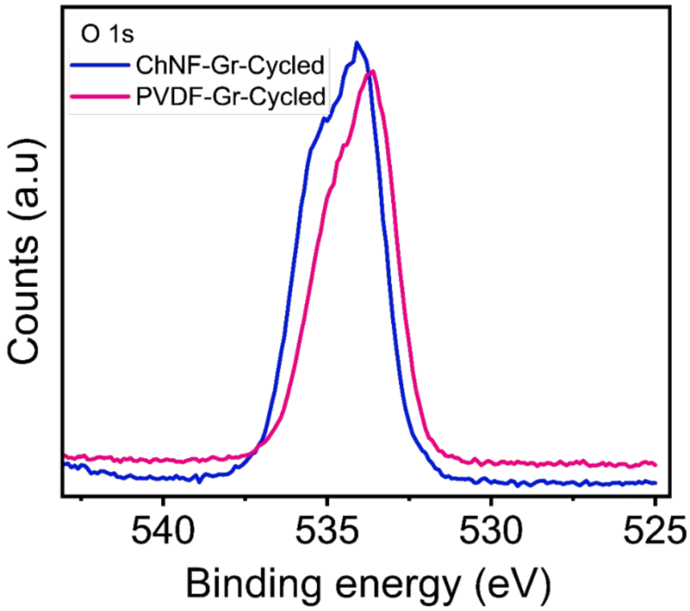

Figure S9: XPS spectra of Oxygen 1s of cycled ChNF-Gr and PVDF-Gr electrodes.

$$H_2O + e^- \longrightarrow OH^- + ½\,H_2 \quad \text{(EQ1)}$$

$$2Li^+ + 2H_2O + 2e^- \longrightarrow 2LiOH + H_2 \quad \text{(EQ2)}$$

$$2LiOH + 2Li^+ + 2e^- \longrightarrow 2Li_2O + H_2 \quad \text{(EQ3)}$$

$$LiPF_6 + H_2O \longrightarrow 2HF + LiF + POF_3 \quad \text{(EQ4)}$$

$$2Li^+ + 2HF + 2e^- \longrightarrow 2LiF + H_2 \quad \text{(EQ5)}$$

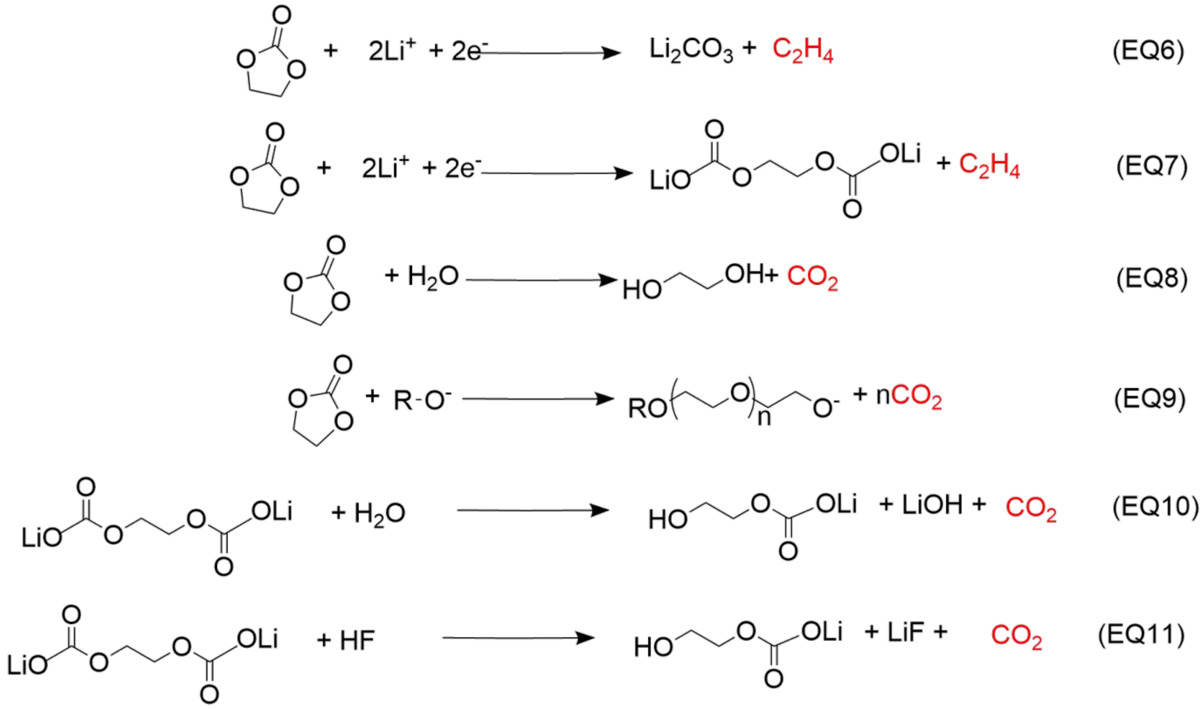

$$R\text{-}OH + LiPF_6 \longrightarrow R\text{-}O\text{-}PF_4 + LiF + HF \quad \text{(EQ12)}$$

Figure S10: Gas evolution reaction mechanisms associated with SEI formation in graphite electrodes.

Table S4: Specific capacities of graphite electrodes with the PVDF and the reported water-based binders.

Binder	Additives	Percentage	Specific capacity (0.1C rate at 25 °C)	Reference
PVDF	-	10	201.9	[1]
PVDF	-	8	387	[2]
PVDF	-	4	342	[3]
PVDF	-	5	337	[4]
PVDF	-	8	364.9	[5]
PVDF	-	10	347	[6]
**Water based binders**				
CMC	SBR	10	341.2	[1]
Na-CMC	-	2	350	[2]
		5	325	
		8	371	
		12	111	
Na-Alginate	-	2	377	[2]
		5	382	
		8	347	
		12	324	
Gum Arabic	-	5	292	[2]
		8	232	
		12	291	
Acryl S020	-	6	355	[7]
Na-CMC	-	10	290	[8]
PAA-Na	-	10	350	[8]
SBR	-	10	350	[8]
Na-CMC	SBR	4	340	[3]
Tragacanth gum	-	4	338	[3]
Chitosan	-	4	333	[3]
Gelatine	-	4	359	[3]

Alginate	-	4	348	[3]
Chitosan	-	6	374	[5]
Polymer gel binder (chitosan ionic liquid)	-		364	[4]
White-Latex	-	5	357	[6]
Chitin nanofibers (this work)		4	370	

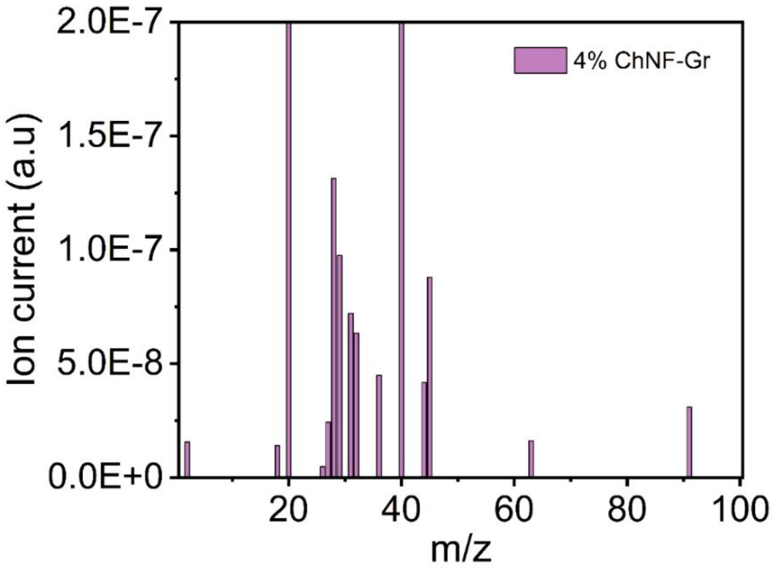

Figure S11: Full scan spectra of PVDF-Gr cell after the formation.

Reference

(1) Azaki, N. J.; Ahmad, A.; Hassan, N. H.; Mohd Abdah, M. A. A.; Su'ait, M. S.; Ataollahi, N.; Lee, T. K. Poly(Methyl Methacrylate) Grafted Natural Rubber Binder for Anodes in Lithium-Ion Battery Applications. *ACS Appl. Polym. Mater.* **2023**, *5* (7), 4953–4965. https://doi.org/10.1021/acsapm.3c00532.

(2) Cuesta, N.; Ramos, A.; Cameán, I.; Antuña, C.; García, A. B. Hydrocolloids as Binders for Graphite Anodes of Lithium-Ion Batteries. *Electrochim. Acta* **2015**, *155*, 140–147. https://doi.org/10.1016/j.electacta.2014.12.122.

(3) Versaci, D.; Nasi, R.; Zubair, U.; Amici, J.; Sgroi, M.; Dumitrescu, M. A.; Francia, C.;

Bodoardo, S.; Penazzi, N. New Eco-Friendly Low-Cost Binders for Li-Ion Anodes. *J. Solid State Electrochem.* **2017**, *21* (12), 3429–3435. https://doi.org/10.1007/s10008-017-3665-5.

(4) Ding, L.; Bagul, P.; Cui, L.; Oswald, S.; Pohle, B.; Leones, R.; Mikhailova, D. Graphite Anode Functionalized with a Gel Biopolymer Binder for Li-Ion Batteries Operating in a Broad Temperature Range. *ACS Appl. Energy Mater.* **2023**, *6* (8), 4404–4412. https://doi.org/10.1021/acsaem.3c00512.

(5) Zhang, L.; Chai, L.; Qu, Q.; Zhang, L.; Shen, M.; Zheng, H. Chitosan, a New and Environmental Benign Electrode Binder for Use with Graphite Anode in Lithium-Ion Batteries. *Electrochim. Acta* **2013**, *105*, 378–383. https://doi.org/10.1016/j.electacta.2013.05.009.

(6) Lahiru Sandaruwan, R. D.; Kuramoto, R.; Wang, B.; Ma, S.; Wang, H. White Latex: Appealing “Green” Alternative for PVdF in Electrode Manufacturing for Sustainable Li-Ion Batteries. *Langmuir* **2022**, *38* (29), 8934–8942. https://doi.org/10.1021/acs.langmuir.2c01115.

(7) Pohjalainen, E.; Sorsa, O.; Juurikivi, J.; Kallio, T. Water-Soluble Acrylate Binder for Graphite Electrodes in Lithium-Ion Batteries. *Energy Technol.* **2016**, *4* (4), 470–472. https://doi.org/10.1002/ente.201500371.

(8) Jeschull, F.; Lacey, M. J.; Brandell, D. Functional Binders as Graphite Exfoliation Suppressants in Aggressive Electrolytes for Lithium-Ion Batteries. *Electrochim. Acta* **2015**, *175*, 141–150. https://doi.org/10.1016/j.electacta.2015.03.072.
